# Supplementary material for: National data meets AI: Machine learning for predicting overweight/obesity among ever-married Bangladeshi women
Source: PLoS One. 2026 Feb 2;21(2):e0341821. doi: 10.1371/journal.pone.0341821 (PMC12863505; doi:10.1371/journal.pone.0341821)
Supplement: S1 Table — (DOCX) [file pone.0341821.s001.docx]

**S1 Table.** Bivariate analysis of factors associated with Underweight/Normal and Overweight/Obese among ever-married women in Bangladesh.

| **Variables** | **Underweight/Normal, n (%)** | **Overweight/Obese, n (%)** | **Total, N (%)** | **Chi-Square** | **P-Value** |
| --- | --- | --- | --- | --- | --- |
| **Age in 5-year groups** | | | | 649.711 | <0.001 |
| 15-19 | 1273 (7.8%) | 153 (0.9%) | 1426 (8.7%) |  |  |
| 20-24 | 2116 (13.0%) | 590 (3.6%) | 2706 (16.6%) |  |  |
| 25-29 | 1935 (11.9%) | 971 (6.0%) | 2906 (17.8%) |  |  |
| 30-34 | 1819 (11.2%) | 1120 (6.9%) | 2939 (18.0%) |  |  |
| 35-39 | 1479 (9.1%) | 1044 (6.4%) | 2523 (15.5%) |  |  |
| 40-44 | 1165 (7.1%) | 777 (4.8%) | 1942 (11.9%) |  |  |
| 45-49 | 1164 (7.1%) | 697 (4.3%) | 1861 (11.4%) |  |  |
| **Division** | | | | 240.024 | <0.001 |
| Barishal | 1191 (7.3%) | 599 (3.7%) | 1790 (11.0%) |  |  |
| Chittagong | 1389 (8.5%) | 905 (5.6%) | 2294 (14.1%) |  |  |
| Dhaka | 1355 (8.3%) | 859 (5.3%) | 2214 (13.6%) |  |  |
| Khulna | 1351 (8.3%) | 826 (5.1%) | 2177 (13.4%) |  |  |
| Mymensingh | 1388 (8.5%) | 453 (2.8%) | 1841 (11.3%) |  |  |
| Rajshahi | 1447 (8.9%) | 690 (4.2%) | 2137 (13.1%) |  |  |
| Rangpur | 1512 (9.3%) | 592 (3.6%) | 2104 (12.9%) |  |  |
| Sylhet | 1318 (8.1%) | 428 (2.6%) | 1746 (10.7%) |  |  |
| **Type of place of residence** | | | | 414.680 | <0.001 |
| Urban | 3143 (19.3%) | 2397 (14.7%) | 5540 (34.0%) |  |  |
| Rural | 7808 (47.9%) | 2955 (18.1%) | 10763 (66.0%) |  |  |
| **Respondent's highest educational level** | | | | 231.290 | <0.001 |
| No education | 1931 (11.8%) | 640 (3.9%) | 2571 (15.8%) |  |  |
| Primary | 3668 (22.5%) | 1590 (9.8%) | 5258 (32.3%) |  |  |
| Secondary | 4149 (25.4%) | 2153 (13.2%) | 6302 (38.7%) |  |  |
| Higher | 1203 (7.4%) | 969 (5.9%) | 2172 (13.3%) |  |  |
| **Source of drinking water** | | | | 32.932 | <0.001 |
| Improved | 10701 (65.6%) | 5299 (32.5%) | 16000 (98.1%) |  |  |
| Unimproved | 250 (1.5%) | 53 (0.3%) | 303 (1.9%) |  |  |
| **Type of toilet facility** | | | | 350.230 | <0.001 |
| Improved | 6866 (42.1%) | 4138 (25.4%) | 11004 (67.5%) |  |  |
| Unimproved | 4085 (25.1%) | 1214 (7.4%) | 5299 (32.5%) |  |  |
| **Household has: electricity** | | | | 300.942 | <0.001 |
| No | 2403 (14.7%) | 576 (3.5%) | 2979 (18.3%) |  |  |
| Yes | 8548 (52.4%) | 4776 (29.3%) | 13324 (81.7%) |  |  |
| **Household has: television** | | | | 718.693 | <0.001 |
| No | 6254 (38.4%) | 1860 (11.4%) | 8114 (49.8%) |  |  |
| Yes | 4697 (28.8%) | 3492 (21.4%) | 8189 (50.2%) |  |  |
| **Household has: refrigerator** | | | | 1051.853 | <0.001 |
| No | 8410 (51.6%) | 2766 (17.0%) | 11176 (68.6%) |  |  |
| Yes | 2541 (15.6% | 2586 (15.9% | 5127 (31.4%) |  |  |
| **Household has: bicycle** | | | | .008 | 0.929 |
| No | 7611 (46.7%) | 3716 (22.8%) | 11327 (69.5%) |  |  |
| Yes | 3340 (20.5%) | 1636 (10.0%) | 4976 (30.5% |  |  |
| **Household has: motorcycle/scooter** | | | | 219.299 | <0.001 |
| No | 10144 (62.2%) | 4565 (28.0%) | 14709 (90.2%) |  |  |
| Yes | 807 (5.0%) | 787 (4.8%) | 1594 (9.8%) |  |  |
| **Household has: car/truck** | | | | 39.998 | <0.001 |
| No | 10887 (66.8%) | 5267 (32.3%) | 16154 (99.1%) |  |  |
| Yes | 64 (0.4%) | 85 (0.5%) | 149 (0.9%) |  |  |
| **Religion** | | | | 7.300 | 0.063 |
| Islam | 9826 (60.3%) | 4821 (29.6%) | 14647 (89.8%) |  |  |
| Hinduism | 1063 (6.5%) | 496 (3.0%) | 1559 (9.6%) |  |  |
| Buddhism | 51 (0.3% | 21 (0.1%) | 72 (0.4%) |  |  |
| Christianity | 11 (0.1%) | 14 (0.1%) | 25 (0.2%) |  |  |
| **Sex of household head** | | | | 5.036 | 0.025 |
| Male | 9751 (59.8%) | 4702 (28.8%) | 14453 (88.7%) |  |  |
| Female | 1200 (7.4%) | 650 (4.0%) | 1850 (11.3%) |  |  |
| **Frequency of watching television** | | | | 516.735 | <0.001 |
| Not at all | 4751 (29.1%) | 1437 (8.8%) | 6188 (38.0%) |  |  |
| Less than once a week | 1028 (6.3%) | 383 (2.3%) | 1411 (8.7%) |  |  |
| At least once a week | 5172 (31.7%) | 3532 (21.7%) | 8704 (53.4%) |  |  |
| **Type of cooking fuel** | | | | 647.596 | <0.001 |
| Solid Fuel | 9477 (58.1%) | 3742 (23.0%) | 13219 (81.1%) |  |  |
| Clean Fuel | 1474 (9.0%) | 1610 (9.9%) | 3084 (18.9%) |  |  |
| **Wealth index combined** | | | | 1326.676 | <0.001 |
| Poorest | 2637 (16.2%) | 544 (3.3%) | 3181 (19.5%) |  |  |
| Poorer | 2482 (15.2%) | 702 (4.3%) | 3184 (19.5%) |  |  |
| Middle | 2233 (13.7%) | 977 (6.0%) | 3210 (19.7%) |  |  |
| Richer | 2035 (12.5%) | 1232 (7.6%) | 3267 (20.0%) |  |  |
| Richest | 1564 (9.6%) | 1897 (11.6%) | 3461 (21.2%) |  |  |
| **Wealth index for urban/rural** | | | | 946.133 | <0.001 |
| Poorest | 2802 (17.2%) | 667 (4.1%) | 3469 (21.3%) |  |  |
| Poorer | 2487 (15.3%) | 838 (5.1%) | 3325 (20.4%) |  |  |
| Middle | 2216 (13.6%) | 994 (6.1%) | 3210 (19.7%) |  |  |
| Richer | 1971 (12.1%) | 1261 (7.7%) | 3232 (19.8%) |  |  |
| Richest | 1475 (9.0%) | 1592 (9.8%) | 3067 (18.8%) |  |  |
| **Total children ever born** | | | | 133.619 | <0.001 |
| No child | 1077 (6.6%) | 311 (1.9%) | 1388 (8.5%) |  |  |
| One child | 2285 (14.0%) | 930 (5.7%) | 3215 (19.7%) |  |  |
| Two child | 2998 (18.4%) | 1772 (10.9%) | 4770 (29.3%) |  |  |
| More than two child | 4591 (28.2%) | 2339 (14.3%) | 6930 (42.5%) |  |  |
| **Currently pregnant** | | | | 4.066 | 0.044 |
| No/Unsure | 10336 (63.4%) | 5092 (31.2%) | 15428 (94.6%) |  |  |
| Yes | 615 (3.8%) | 260 (1.6%) | 875 (5.4%) |  |  |
| **Current contraceptive method** | | | | 18.057 | <0.001 |
| Not using | 3951 (24.2%) | 1921 (11.8%) | 5872 (36.0%) |  |  |
| Modern methods | 5901 (36.2%) | 2779 (17.0%) | 8680 (53.2%) |  |  |
| Traditional methods | 1099 (6.7%) | 652 (4.0%) | 1751 (10.7%) |  |  |
| **Currently breastfeeding** | | | | 344.711 | <0.001 |
| No | 8256 (50.6%) | 4704 (28.9%) | 12960 (79.5%) |  |  |
| Yes | 2695 (16.5%) | 648 (4.0%) | 3343 (20.5%) |  |  |
| **Husband/partner's education level** | | | | 465.805 | <0.001 |
| No education | 2695 (16.5%) | 907 (5.6%) | 3602 (22.1%) |  |  |
| Primary | 3815 (23.4% | 1447 (8.9%) | 5262 (32.3%) |  |  |
| Secondary | 3027 (18.6%) | 1696 (10.4%) | 4723 (29.0%) |  |  |
| Higher | 1397 (8.6% | 1291 (7.9%) | 2688 (16.5%) |  |  |
| Don’t know | 17 (0.1% | 11 (0.1%) | 28 (0.2%) |  |  |
| **Husband/partner's occupation** | | | | 437.689 | <0.001 |
| Unemployed | 263 (1.6%) | 148 (0.9%) | 411 (2.5%) |  |  |
| Agriculture | 3207 (19.7%) | 1044 (6.4%) | 4251 (26.1%) |  |  |
| Business | 2029 (12.4%) | 1401 (8.6%) | 3430 (21.0%) |  |  |
| Labour/Service | 5018 (30.8% | 2249 (13.8% | 7267 (44.6%) |  |  |
| Job | 434 (2.7% | 510 (3.1% | 944 (5.8%) |  |  |
| **Respondent's occupation** | | | | 208.341 | <0.001 |
| Unemployed | 4957 (30.4%) | 2853 (17.5%) | 7810 (47.9%) |  |  |
| Agriculture | 4274 (26.2% | 1545 (9.5%) | 5819 (35.7%) |  |  |
| Business | 170 (1.0%) | 98 (0.6%) | 268 (1.6%) |  |  |
| Labour/Service | 1383 (8.5%) | 679 (4.2%) | 2062 (12.6%) |  |  |
| Job | 167 (1.0%) | 177 (1.1%) | 344 (2.1%) |  |  |
| **Person who usually decides on respondent's health care** | | | | 50.806 | <0.001 |
| Respondent alone | 1021 (6.3%) | 559 (3.4%) | 1580 (9.7%) |  |  |
| Respondent & husband/partner | 9357 (57.4%) | 4640 (28.5%) | 13997 (85.9%) |  |  |
| Respondent & other person | 573 (3.5%) | 153 (0.9%) | 726 (4.5%) |  |  |
