# Supplementary material for: National data meets AI: Machine learning for predicting overweight/obesity among ever-married Bangladeshi women
Source: PLoS One. 2026 Feb 2;21(2):e0341821. doi: 10.1371/journal.pone.0341821 (PMC12863505; doi:10.1371/journal.pone.0341821)
Supplement: S2 Table — (DOCX) [file pone.0341821.s002.docx]

**S2 Table**. Feature selection by Filter, Wrapper, and Embedded methods

| **Filter method (Chi-square)** | **Wrapper method (SFS)** | **Embedded method (LASSO)** |
| --- | --- | --- |
| Wealth index, Household has refrigerator, Type of cooking fuel, Frequency of watching television, age, Household has television, partner's education, Currently breastfeeding, Household has motorcycle, Division, Highest educational level, Type of toilet facility, Type of place of residence, Household has electricity, Partner's occupation, Household has: car/truck, Total children ever born, and Currently pregnant | Age, Division, Type of place of residence, Highest educational level, Partner's education, Sex of household head, Total children ever born, Household has bicycle, Household has electricity, Household has refrigerator, Household has television, Frequency of watching television, Occupation, Partner’s occupation, Wealth index, Wealth index for urban/rural, Source of drinking water, Type of toilet facility, Type of cooking fuel, Currently pregnant, Currently breastfeeding, Current contraceptive method | Age, Division, Highest educational level, Partner's education, Total children ever born, Household has refrigerator, Frequency of watching television, Partner's occupation, Wealth index, Type of cooking fuel, and Currently breastfeeding |
