# Supplementary figures and images for: National data meets AI: Machine learning for predicting overweight/obesity among ever-married Bangladeshi women
Source: PLoS One. 2026 Feb 2;21(2):e0341821. doi: 10.1371/journal.pone.0341821 (PMC12863505; doi:10.1371/journal.pone.0341821)

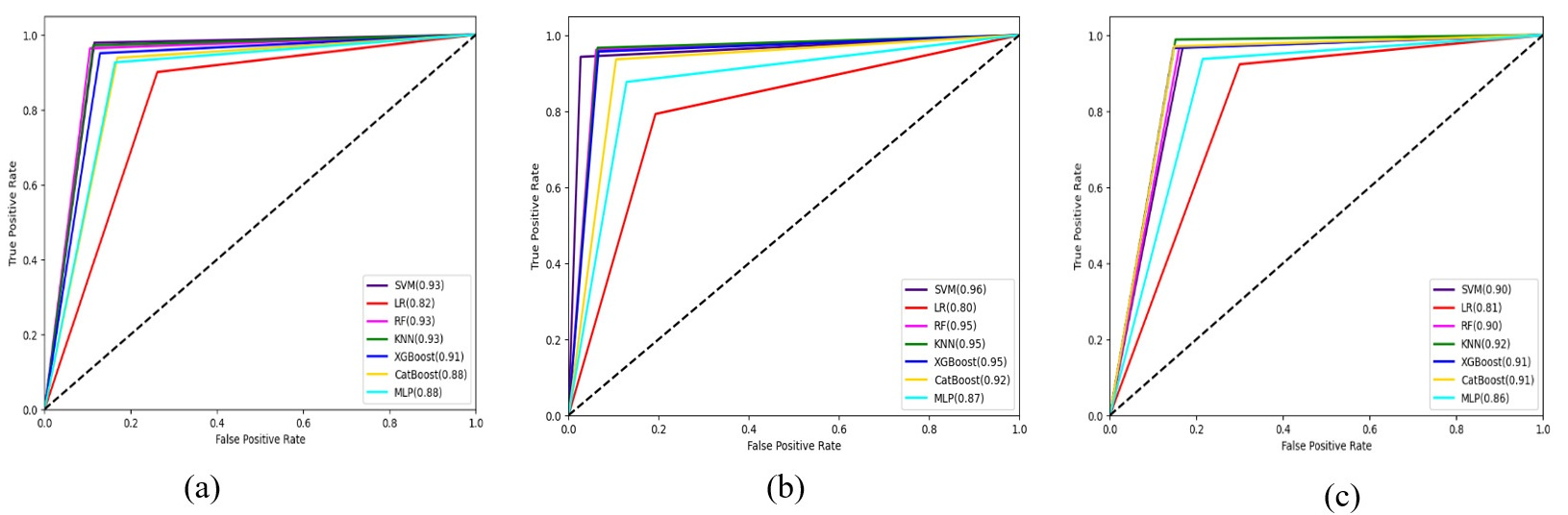

Supplement: S1 Fig — (TIF) [file pone.0341821.s003.tif]
